# No association between the common calcium-sensing receptor polymorphism rs1801725 and irritable bowel syndrome

**DOI:** 10.1186/s12881-015-0256-0

**Published:** 2015-12-11

**Authors:** Philipp Romero, Stefanie Schmitteckert, Mira M. Wouters, Lesley A. Houghton, Bastian Czogalla, Gregory S. Sayuk, Guy E. Boeckxstaens, Patrick Guenther, Stefan Holland-Cunz, Beate Niesler

**Affiliations:** Department of Surgery, Division of Paediatric Surgery, University of Heidelberg, Heidelberg, Germany; Department of Human Molecular Genetics, Institute of Human Genetics, University of Heidelberg, Im Neuenheimer Feld 366, Heidelberg, 69120 Germany; TARGID, University Hospital Leuven, Leuven, Belgium; University of Manchester, Manchester, UK & Mayo Clinic, Jacksonville, USA; Washington University School of Medicine, St. Louis, USA; Department of Paediatric Surgery, University of Basel, Basel, Switzerland

**Keywords:** Calcium-sensing receptor, Irritable bowel syndrome, Gut motility

## Abstract

**Background:**

The calcium-sensing receptor (CaSR) is a calcium (Ca^2+^) sensitive G protein-coupled receptor implicated in various biological processes. In particular, it regulates Ca^2+^/Mg^2+^- homeostasis and senses interstitial Ca^2+^ levels and thereby controls downstream signalling cascades. Due to its expression in the gut epithelium, the enteric nervous system and smooth muscles and its key function in regulation and coordination of muscular contraction and secretion, it represents an excellent candidate gene to be investigated in the pathophysiology of irritable bowel syndrome (IBS). Disturbed CaSR structure and function may impact gastrointestinal regulation of muscular contraction, neuronal excitation and secretion and consequently contribute to symptoms seen in IBS, such as disordered defecation as well as disturbed gut motility and visceral sensitivity.

**Methods:**

We have therefore genotyped the functional *CASR* SNP rs1801725 in three case control samples from the UK, Belgium and the USA.

**Results:**

Genotype frequencies showed no association in the three genotyped case–control samples, neither with IBS nor with IBS subtypes.

**Conclusions:**

Although we could not associate the SNP to any of the established bowel symptom based IBS subtypes we cannot rule out association to altered Ca^2+^ levels and disturbed secretion and gut motility which were unfortunately not assessed in the patients genotyped. This underlines the necessity of a more detailed phenotyping of IBS patients and control individuals in future studies.

## Background

The calcium-sensing receptor (CaSR) is a multifunctional G protein-coupled receptor implicated in various biological processes [1, 2]. Besides its expression in tissues maintaining systemic calcium/magnesium (Ca^2+^/Mg^2+^) homeostasis such as parathyroid, thyroid glands and the kidney, it can also be found in the gastrointestinal (GI) tract. The receptors have been detected in the gut epithelium from the stomach to the small and large intestine [3, 4], as well as in the plexus submucosus and plexus myentericus of the enteric nervous system (ENS) [5] and in smooth muscle cells [6]. To date, the CaSR has been shown to be involved in the regulation of colonic secretion [7, 8] and modulation of gut motility in response to changes of interstitial Ca^2+^ levels [9–11]. Interestingly, studies have shown that constipation in hyperparathyroidism is associated with reduced neuromuscular excitability as a result of increased interstitial Ca^2+^ levels [12]. This was mediated by CaSRs on the myenteric plexus regulating smooth muscle function and coordination [10, 11]. Intestinal motility is modulated in a highly complex manner with Ca^2+^ playing a fundamental role in regulation of muscular contraction and neuronal excitation. Contraction of smooth muscle cells is controlled by the interstitial Ca^2+^ levels and the sensitivity of the contractile elements to Ca^2+^ in response to changes in the interstitial space [13]. Activation of CaSRs causes Ca^2+^ release from intracellular Ca^2+^ stores such as the endoplasmic reticulum by initializing intracellular second messenger cascades and consequently increasing interstitial Ca^2+^ levels [6, 14]. Consequently, a dysfunctional CaSR causing disturbed intestinal Ca^2+^ absorption may have effects on intestinal motility, secretion [7, 8], neuromuscular excitability, colonic fluid transport, peptide release and contribute to the symptoms seen in functional GI disorders, such as Irritable Bowel Syndrome (IBS).

IBS patients present with abdominal pain associated with a change in bowel habit [15]. Depending on predominant bowel habit, IBS is further sub-typed into IBS-C (constipation), IBS-D (diarrhea), IBS-M (mixed constipation/ diarrhea) and IBS-U (unspecified) [16–21]. Despite intense research, its pathophysiology remains unclear but is thought in part to be related to disordered motility, secretion and visceral sensation. Dysfunction within the ENS [22] and/or central nervous system, along with impaired mucosal barrier function have all been implicated in its pathophysiology [23].

Interestingly, a large meta-analysis including 12.865 individuals of European and Indian descent correlated serum Ca^2+^ level with single nucleotide polymorphisms (SNPs) in the *CASR* gene [24]. In particular, the minor allele of the *CASR* SNP rs1801725 was reported to be strongly associated with increased serum Ca^2+^ levels (*p* < 0.001). This SNP encodes a missense variant which leads to a non-conservative amino acid change: (c.2956 G > T, p.Ala986Ser = A986S) residing in the cytoplasmic tail of the CaSR [24]. *In vitro* studies showed that mutations within the respective region of the receptor may influence CaSR function, impair signal transduction, intracellular trafficking and/or cell surface expression, respectively [25–27]. The functions and influences of a dysfunctional CaSR have not been explored in IBS previously. We hypothesized that this common *CASR* polymorphism may be relevant in the pathophysiology of IBS and evaluated rs1801725 in patients with IBS from three case–control cohorts from the UK, Belgium and the USA.

## Methods

### Genotyped study subjects

#### Case–control cohort from Manchester (UK)

SNP genotyping was performed in a cohort of 86 patients with IBS-D (aged 18–66 years; mean age 41.7 years; 60 female, 26 male), 88 with IBS-C (aged 18–65 years; mean age 39.9 years; 82 female, 6 male) and 86 healthy controls (aged 18–63 years; mean age 35.0 years; 55 female, 31 male) (Table 1). IBS patients with mixed bowel habit (IBS-M) were not included. Recruitment of IBS patients was via the Out Patients Departments of the University Hospital of South Manchester, local general practices, advertisement in regional newspapers and an existing departmental volunteer pool of patients. All patients satisfied the Rome II criteria for IBS and predominant bowel habit subtype [28]. Moreover, they underwent appropriate investigations to exclude organic disease [15] and did not show any functional disorder of the upper GI tract that was more prominent than their IBS. In addition, no subject had a history of major psychiatric disorder or history of alcohol or substance abuse. Healthy controls were recruited by advertisement. All subjects were Caucasian and drank below the recommended safe alcohol limit (<21 units/week) smoked <5 cigarettes per day. Written consent was obtained from all subjects and the study was approved by the South Manchester Medical Research Ethics Committee. DNA was extracted from peripheral blood cells taken from both the patients and healthy controls using standard protocols [29].

**Table 1:** Characteristics of the genotyped case–control cohorts

case–control cohort	IBS patients	Sex	Age	IBS-C patients	IBS-D patients	healthy volunteers	Sex	Age
UK	174	♀142 ♂32	18-66 years ø 40.8 years	88	86	86	♀55 ♂31	18-63 years ø 35.0 years
USA	44	♀28 ♂16	20-83 years ø 47.9 years	16	28	86	♀45 ♂41	18-81 years ø 55.9 years
BEL	733	♀593 ♂140	18-83 years ø 42.3 years	313	420	622	♀470 ♂152	18-82 years ø 43.5 years
Total	951	♀819 ♂200	18-83 years ø 43.6 years	417	534	794	♀570 ♂224	18-82 years ø 44.8 years

#### Case–control cohort from St. Louis, Missouri (USA)

The second genotyped cohort included 28 patients with IBS-D (aged 20–83 years; mean age 49.9 years; 18 female, 10 male), 16 IBS-C patients (aged 28–64 years; mean age 45.9 years; 10 female, 6 male); and 86 healthy controls (aged 18–81 years; mean age 55.9 years; 45 female, 41 male) (Table 1). IBS patients with mixed bowel habit (IBS-M) were not included. The group was composed of adult subjects (≥18 years old) who carried a clinical functional GI disease (FGID) diagnosis, and met Rome III criteria for IBS. Prior to enrolment in the study, all FGID subjects had organic bowel disease excluded with a comprehensive evaluation, including but not limited to laboratory, endoscopic and radiographic testing, performed at the discretion of the treating gastroenterologist. The control group consisted of patients undergoing screening endoscopy in the absence of on going GI diagnoses or symptoms at the time of enrolment, and without evidence for FGID using Rome III criteria. All participants provided written informed consent prior to collection of biological specimens and clinical data. The study protocol was approved by the Human Research Protection Office (institutional review board) at Washington University School of Medicine and Barnes-Jewish Hospital, St. Louis, Missouri. Sputum and/or blood samples were collected for DNA analysis at the time of subject enrolment. Blood was collected in cases where the participant was to undergo phlebotomy for clinical purposes. If a blood specimen was not required for other laboratory testing, a sputum sample was collected. DNA was extracted using Qiagen Gentra Puregene Reagents (Qiagen Inc., Valencia, CA). Following DNA extraction, samples were frozen and banked by the Biobank Core of the Washington University Digestive Diseases Research Center.

#### Case–control cohort from Leuven (Belgium, BEL)

The third genotyped cohort included 420 patients with IBS-D (aged 18–83 years; mean age 41.0 years; 305 female, 115 male), 313 IBS-C patients (aged 18–82 years; mean age 43.6 years; 288 female, 25 male) and 622 healthy controls (aged 18–82 years; mean age 43.5 years; 470 female, 152 male) (Table 1). IBS patients with mixed bowel habit (IBS-M) were not included. Further details can be found in Wouters et al. [30].

### Genotyping

The SNP genotyping was performed with the KASPar® assay system (KBiosciences, Ltd, Hoddesdon, UK) as recommended by the manufacturer using a customized assay. An initial 15-min cycle at 94 °C was followed by 20 cycles consisting of 10 s at 94 °C, 5 s at 57 °C, and 10 s at 72 °C; 28 cycles consisting of 10 s at 94 °C, 20 s at 57 °C, and 40 s at 72 °C; and a cool down at 10 °C. After the thermal cycling, KASPar® analysis was performed using the fluorescence plate reader TaqMan7500 system (Applied Biosystems, Foster City, CA).

### Statistical analysis

Chi-squared tests were used to assess deviations of observed genotype frequencies from Hardy-Weinberg-Equilibrium (HWE). Genotype relative risks of IBS were quantified by odds ratios (ORs) with corresponding 95 % confidence intervals (CIs) based on a logistic regression model under additive genetic penetrance. Tests for deviation from Hardy-Weinberg equilibrium and tests for association were performed using an online tool provided by the Institute of Human Genetics, Technical University of Munich (http://ihg.gsf.de/cgi-bin/hw/hwa1.pl). In addition, results were stratified by IBS subtypes. Probability values were adjusted for multiplicity using the Holm-Bonferroni method.

## Results

In our study we analyzed the SNP rs1801725 (p. A986S) of the *CASR* gene, which has not been examined in IBS previously. We investigated three case–control samples with 1745 individuals of Caucasian origin (Table 1). Genotype frequencies did not deviate from Hardy-Weinberg equilibrium and showed no association in the three genotyped case–control samples, neither with IBS nor with IBS subtypes (Table 2). In addition, no significant differences between the three case–control cohorts could be identified.

**Table 2:** Genotyping data for *CASR* p. A986S (rs1801725) in the three case–control cohorts

		IBS			IBS-C	IBS-D
case–control cohort	SNP	OR (95 % CI)	Holm-Bonferroni adjusted P-value	P-value HWE	OR (95 % CI)	OR (95 % CI)
Total	rs1801725	0.95 (0.81-1.19)	0.85	0.49	0.97 (0.79-1.29)	0.92 (0.76-1.20)
UK	rs1801725	1.257 (0.67-1.93)	0.63	0.23	1.041 (0.54-1.811)	1.481 (0.726-2.37)
USA	rs1801725	1.12 (0.51-2.92)	0.65	0.3	1.122 (0.32-3.22)	0.851 (0.25-1.96)
BEL	rs1801725	1.014 (0.78-1.21)	0.84	1.0	1.015 (0.74-1.29)	1.013 (0.76-1.25)

## Discussion

This is the first study to investigate whether the *CASR* SNP rs1801725 associates with the functional GI disorder IBS. The investigated SNP is known to be associated with increased serum Ca^2+^ levels [24] and has therefore a potentially inactivating effect on the CaSR. The set point (= onset of activation) is increased for inactivating mutations or polymorphisms compared to *CASR* wild-type. For receptor activation, the concentration of the CaSR agonist Ca^2+^ needs to be higher in comparison to the CaSR wild-type. Consequently, with respect to intestinal motility, an increased frequency of the *CASR* p.986S allele in the IBS-D group compared to control individuals would be conceivable. Although our study showed no association overall or in any of the three genotyped cohorts or with respect to the established bowel symptom based IBS-subtypes, association to changed motility and transit cannot be excluded. In fact, disturbances in the motility of the GI tract have been shown to be an important feature of IBS, with these varying with sub-type studied. In this sense, IBS-D patients have been shown to have increased colonic motility, particularly high amplitude propagating contractions (HAPCs) and accelerated transit [31]. In contrast, IBS-C patients exhibit reduced motility, less HAPCs and slower transit compared with healthy volunteers [32]. Furthermore, IBS-D patients presented with an increased basal motility index (contractions per minute) compared to patients with IBS-C [33]. Although in general these various motor patterns associate with a particular sub-type of IBS, not all IBS patients in those subtypes will exhibit them. Stratification for any of these traits would have increased the likelihood to correlate a functional SNP with in a particular subset of patients. However, additional analysis stratifying for total serum Ca^2+^ levels, disturbed motility or secretion were not possible since this data were lacking. This underlines the importance of a more detailed patient characterization and assessment of relevant traits and laboratory parameters. Colonic motility for example could be assessed by oroanal transit time measurement [34, 35]. Stool scale form could be taken into account as measure for transit time implementing the Bristol Stool Scale into the phenotyping pipeline of the patients and control individuals [36].

## Conclusions

Although we could not associate the SNP to any of the established bowel symptom based IBS subtypes we cannot rule out association to altered Ca ^2+^ levels and gut motility which were unfortunately not assessed in the patients genotyped. Further investigation of *CASR*-related genetic variations in IBS and relevant intermediate traits such as Ca^2+^ serum levels in addition to the assessment of motility, transit and secretion are needed for a detailed characterization of the gene function and a better understanding of the correlation between *CASR* variants and the aetiology of IBS.
